# Radical esophagectomy for a 92-year-old woman with granulocyte colony-stimulating factor-producing esophageal squamous cell carcinoma: a case report

**DOI:** 10.1186/s12957-016-1023-1

**Published:** 2016-10-13

**Authors:** Mari Kitani, Yukinori Yamagata, Asami Tanabe, Kouichi Yagi, Susumu Aikou, Takashi Kiyokawa, Masato Nishida, Hiroharu Yamashita, Kazuhiko Mori, Sachiyo Nomura, Yasuyuki Seto

**Affiliations:** 1 Department of Gastrointestinal Surgery, The University of Tokyo Hospital, Tokyo, Japan; 2 Department of Surgery, Dokkyo Medical University Koshigaya Hospital, 2-1-50 Minami-Koshigaya, Koshigaya City, Saitama Japan

**Keywords:** Granulocyte colony-stimulating factor, Esophageal squamous cell carcinoma, Granulocyte colony-stimulating factor-producing esophageal squamous cell carcinoma

## Abstract

**Background:**

Granulocyte colony-stimulating factor (G-CSF)-producing esophageal squamous cell carcinoma (ESCC) has been considered to have a poor prognosis. We successfully treated a case of G-CSF-producing ESCC in a 92-year-old woman.

**Case presentation:**

A 92-year-old woman was admitted to our hospital with the complaints of choking while swallowing and dysphagia. Esophagogastroduodenoscopy and contrast-enhanced computed tomography revealed a type 2 esophageal cancer located 26–35 cm from the dental arch, with no distant metastasis. The patient was diagnosed with G-CSF-producing ESCC based on remarkable leukocytosis and high G-CSF levels. The patient underwent radical subtotal esophagectomy. Subsequently, the level of neutrophils (from 23,500/μL to 5000/μL) and the level of G-CSF (from 131 to <19.5 pg/mL) decreased significantly. Immunohistochemistry analysis of the resected tissue specimen showed positive staining for G-CSF in the cytoplasm of the tumor cells. Although the patient developed aspiration pneumonitis, after antibiotic treatment, she promptly recovered and was discharged.

**Conclusions:**

Herein, we describe a case of successfully treated G-CSF-producing ESCC in a 92-year-old woman. Precise detection and safely performed immediate radical operation are considered essential to achieve a good clinical course.

## Background

In addition to the mass tumor effects, granulocyte colony-stimulating factor (G-CSF)-producing tumors display additional signs and symptoms of inflammation caused by G-CSF-producing malignant cells [1]. There have been a relatively high number of reports on G-CSF-producing lung carcinoma; however, reports on G-CSF-producing esophageal squamous cell carcinoma (ESCC) have been scarce.

With the aging of the population, the number of oldest old patients with cancer comorbidities has been increasing [2]. Therefore, effort should be made to determine the effectiveness of each treatment plan.

We report a very rare case of a 92-year-old woman who was promptly diagnosed with G-CSF-producing ESCC and successfully underwent surgical treatment.

## Case presentation

A 92-year-old woman had a major complaint of choking when swallowing or dysphagia. The patient had been healthy and had no particular medical history besides cataract surgery. She had no history of oral medications, smoking, or alcohol. Another physician had previously attended to her was complaint of choking when swallowing. A narrowing of the lumen of the intrathoracic esophagus was detected by esophagogastroduodenoscopy, and the patient was referred to our hospital for detailed examination.

On admission, abnormal symptoms such as fever, anemia, or jaundice were not detected and the performance status was good (score 0 according to the Eastern Cooperative Oncology Group). Laboratory data on admission showed remarkable leukocytosis (leukocytes 23,500/μL, neutrophils 86.1 %, and no blast cells) and slight decrease in the serum albumin (3.5 g/dL) and C-reactive protein (CRP) levels (1.5 mg/dL). The levels of tumor markers, squamous cell carcinoma antigen (SCC-A), and p53 antibody were high (SCC-A, 3.4 ng/mL; p53, 22.2 U/mL). The respiratory functions and electrocardiograms were within normal ranges. However, the renal function was a slight concern.

**Fig. 1:**
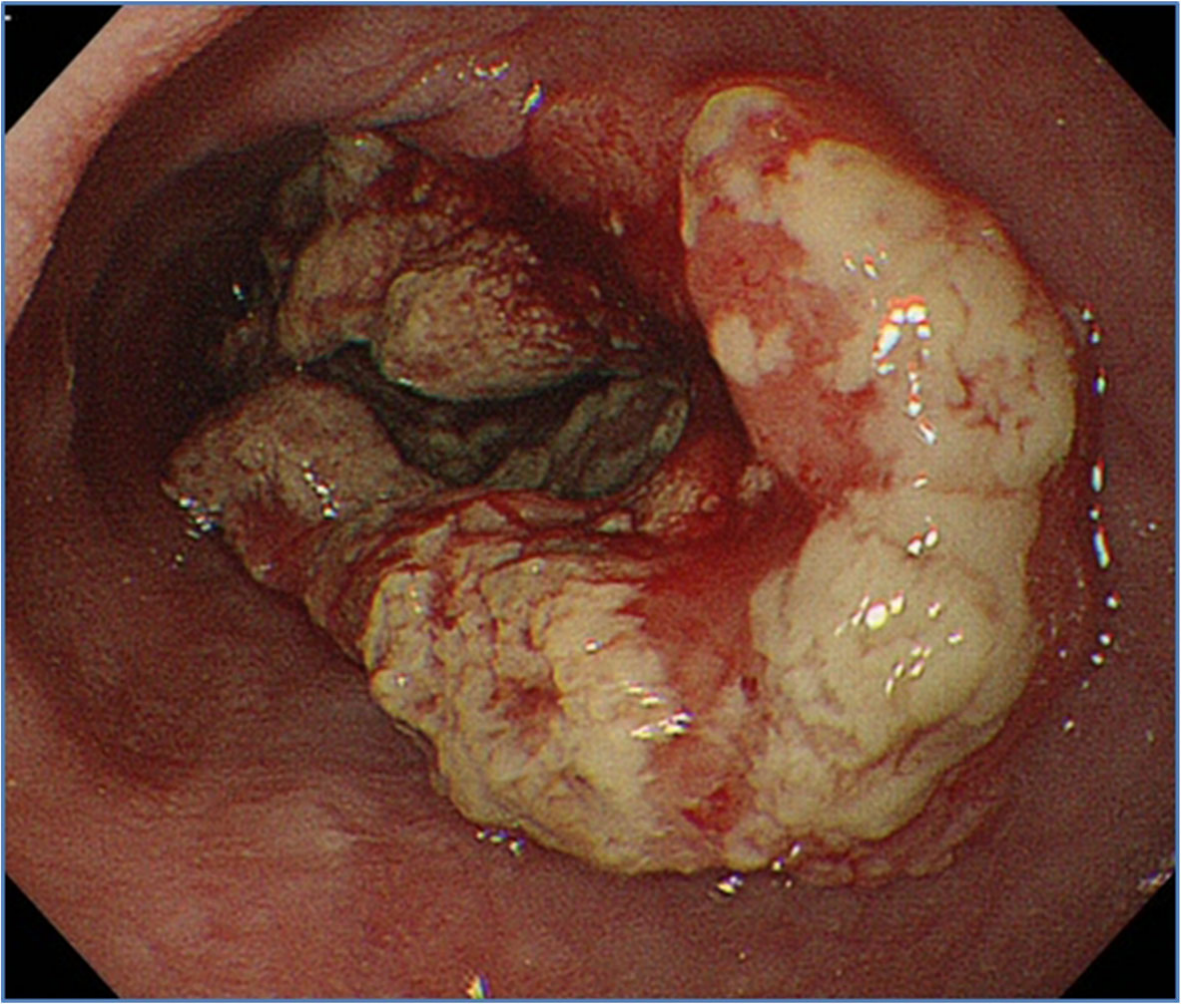
Esophagogastroduodenoscopy findings. Esophagogastroduodenoscopy revealed a type 2, circumferential esophageal cancer of the esophagus approximately at 26–35 cm from the dental arch

**Fig. 2:**
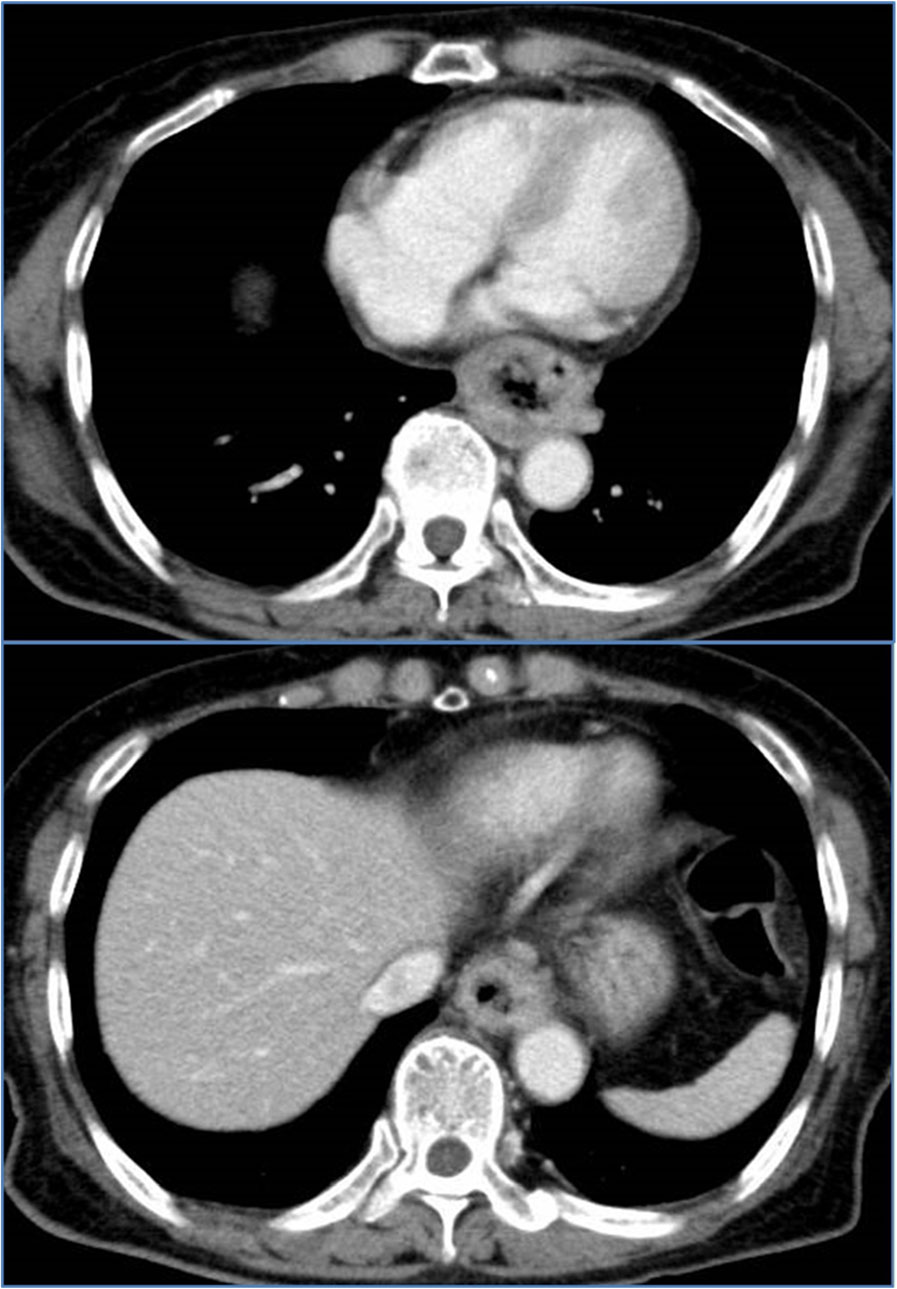
Contrast-enhanced computed tomography findings. Contrast-enhanced computed tomography demonstrated circumferential thickening of the wall of the middle and lower intrathoracic esophagus

Esophagogastroduodenoscopy revealed a type 2, circumferential cancer of the esophagus, approximately at 26–35 cm from the dental arch (Fig. 1), and the biopsy showed SCC. Contrast-enhanced computed tomography of the chest and abdomen demonstrated circumferential thickening of the wall and narrowing of the lumen of the middle and lower intrathoracic esophagus, and small lymph nodes were detected between the lower mediastinum and paracardiac area. Pleural effusion and ascites or distant metastases were not detected (Fig. 2).

Based on these findings, the patient was diagnosed with T3N0M0, stage IIA (according to the Union for International Cancer Control TNM classification of malignant tumors, 7th edition) ESCC. Furthermore, the laboratory data suggested G-CSF-producing carcinoma with serum G-CSF levels of 131 pg/mL.

Despite her age, the patient had no comorbidities, and most importantly, she consented to a surgical operation. Therefore, we planned to perform esophagectomy. In Japan, the standard treatment for stage IIA esophageal carcinoma is subtotal esophagectomy with three-field lymph node dissection following preoperative chemotherapy [3]. However, considering the age disadvantage, multimodal management of disease with chemotherapy or radiotherapy was not performed.

In fact, the subtotal esophagectomy under the right thoracolaparotomy, right lower partial lobectomy, two-field lymph node dissection (instead of three-field), posterior mediastinal route gastric tube reconstruction, and intra-pleural anastomosis were successfully performed. The operation lasted 4 h and 15 min, and the blood loss was 50 mL. The tumor and the right lobe of the lung were attached; therefore, they were resected en bloc because the tumor was considered infiltrative.

**Fig. 3:**
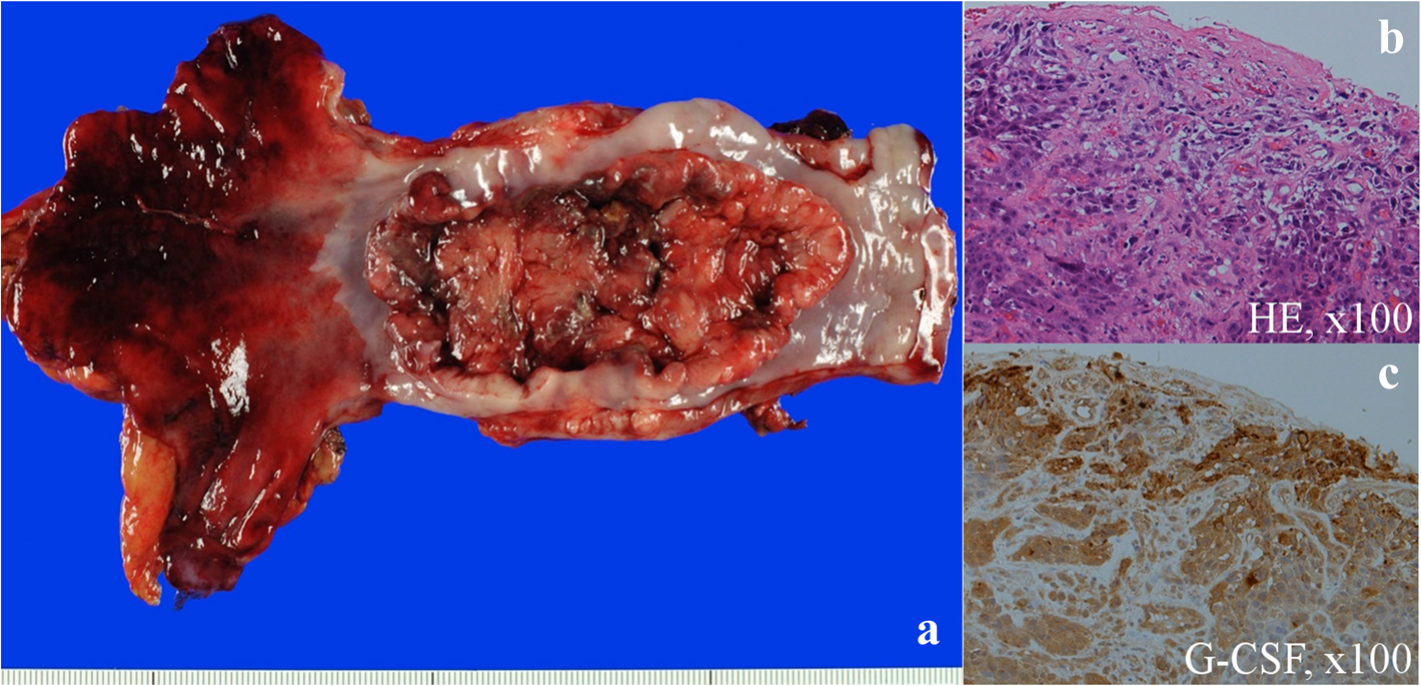
Pathological findings. **a** Macroscopic examination of the primary lesion of the resected esophagus sized 92 × 54 mm. **b** Hematoxylin and eosin stains of the resected tissue specimen revealed moderately differentiated squamous cell carcinoma. **c** Immunohistochemistry of the resected tissue specimen showed positive staining for granulocyte colony-stimulating factor (G-CSF) in the cytoplasm of the tumor cells

Histopathological examination of the resected specimen revealed that the primary lesion sized 92 × 54 mm was a moderately differentiated squamous cell carcinoma with two lymph node metastases, and it was diagnosed as a stage III tumor (according to the Union for International Cancer Control TNM classification) (Fig. 3a, b). Immunohistochemistry of the resected tissue specimen stained positive for G-CSF in the cytoplasm of the tumor cells (Fig. 3c).

After the operation, the patient developed aspiration pneumonitis; however, she promptly recovered with the administration of antibiotics. Three weeks after the operation, the leukocyte counts decreased to 5000/μL and the G-CSF levels to <19.5 pg/mL. Thereafter, the patient exhibited a good clinical course and she was discharged on the 29th postoperative day.

The patient had neutrophilia without any signs of infection or myeloblast genesis before the operation. After esophagectomy, the number of leukocytes and the level of G-CSF had decreased significantly and the presence of G-CSF was confirmed pathologically. Therefore, the patient was definitively confirmed to have a G-CSF-producing tumor.

There have not been any complaints or recurrence, and the patient has remained disease-free from 18 months after the operation until the present day.

### Discussion

Robinson first described a G-CSF-producing tumor in 1974 [1], and the number of such cases has been increasing in the recent years. The primary sites of G-CSF cases have been reported as the lung, urinary tract, or the stomach [4–6]; however, reports of G-CSF-producing esophageal carcinoma have been scarce.

G-CSF is a hematopoietic factor produced by the endothelium, macrophages/monocytes, and fibroblasts. It stimulates the bone marrow to produce granulocytes from stem cells and release neutrophils into the bloodstream [7]. It is also produced by the malignant cancer cells. An excess amount of aberrant production causes an inflammatory response such as fever and positive CRP, a kind of leukemoid reaction (leukocytosis >50,000 leukocytes/μL), and paraneoplastic syndrome in clinical oncology. A recombinant form of G-CSF is currently used to prevent infections after chemotherapy or radiological therapy, which causes myelosuppression and neutropenia.

The diagnostic criteria for G-CSF-producing tumors include (1) a marked increase in the leukocyte counts, (2) elevated G-CSF activity, (3) a decrease in leukocyte counts following tumor resection, and (4) the verification of G-CSF production in the tumor [1]. Because all four criteria were fulfilled, we diagnosed the patient with G-CSF-producing ESCC.

Esophageal carcinoma is a disease with a poor prognosis [8]. Furthermore, the prognosis of G-CSF-producing ESCC is considered even poorer (Table 1) [9–18]. All of the cases have been found at rather advanced stage, in 12 cases (including our case), and 9 cases were poor prognosis. The reason might include (1) G-CSF per se having a capacity to expand tumor growth in an autocrine manner, (2) acute renal failure or hyperuricemia (so-called tumor lysis syndrome) by cytolysis of increased neutrophils after chemotherapy, (3) thrombosis by platelet aggregation by G-CSF [19]. The surviving three patients had undergone tumor resection. Furthermore, among the poor prognosis group, the survival period of excised cases was estimated to be longer than that of non-excised cases. From the above, in cases of the G-CSF-producing ESCC, if possible, the complete tumor resection is considered to be important. Since the prognosis of this disease is much poor, if possible, surgery as well, multimodal therapy that combines radiotherapy and/or chemotherapy is considered preferable.

**Table 1:** Summary of reported cases of G-CSF-producing esophageal squamous cell carcinoma

Case	Author	Age (years)	Gender	Leukocyte (μL)	Serum G-CSF (pg/mL)	Tumor location	Stage (TNM 7th)	Histologic grade (TNM 7th)	History of cancer	Treatment	Prognosis	
1	Watanabe	81	Woman	22,100	1175	Mt	Unknown	Unknown	Lung (simultaneous)	BSC	12 days	Dead
2	Ichiishi	66	Man	33,900	Unknown	Lt	Unknown	G2-3	Stomach (simultaneous)	BSC	2 months	Dead
3	Matsumoto	66	Man	41,500	154	Lt	IV	G2	None	Resection + CRT	l6 months	Dead
4	Kato	54	Man	16,900	150	Lt	IV	G2	None	Chemotherapy	3 months	Deed
5	Komatsu	73	Man	45,710	231	AeLtG	IIB	G2	Stomach (simultaneous)	Resection	19 months	Alive
6	Nakata	56	Man	24,300	78	Lt	IIB	G2	None	Resection + CRT	10 months	Alive
7	Mimatsu	69	Man	19,600	113	Mt	IV	G3	None	Radiation	7 months	Dead
8	Tanabe	76	Man	24,260	134	LtAeG	Unknown	G2	None	Resection, CRT	10 months	Dead
9	Mayanagi	30	Man	19,020	53.7	Mt	IIIC	G1	Leukemia (metachronous)	Neoadjuvant CRT + resection	3 months	Recurrence
10	Shimakawa	73	Man	Unknown	41	Unknown	Unknown	G2	Unknown	Chemotherapy	2 months	Dead
11	Shimakawa	70	Man	16,700	254	Lt	IIIB	G2	None	NAC + resection	12 months	Dead
12	Our case	91	Woman	23,500	131	LtMt	IIIA	G2	None	Resection	18 months	Alive

*Mt* middle intrathoracic esophagus, *Lt* lower intrathoracic esophagus, *Ae* abdominal esophagus, *G* stomach, *BSC* best supporting care, *CRT* chemoradiotherapy, *NAC* neoadjuvant chemotherapy

According to Table 1, G-CSF-producing ESCC was male-dominated (83.3 %) and the average age of the 12 patients was 67 years old. These findings were considered to overlap with the population of normal ESCC. Association between leukocyte value, serum G-CSF value, tumor location, tumor stage, histologic grade, and prognosis was not clear. In addition, in one third in these 12 cases, a merger of other organs’ tumor was observed. It is suggested that the characteristic of G-CSF, which was mentioned above, might have influence on tumor growth [19].

In addition, with the aging of the population, the chances that we encounter the oldest old patients are increasing [8]. The appropriate evaluation of overall conditions and the selection of operative method are critical. The operative reports of the elderly are few, and among those cases, the cytoreductive (limited) operations were often chosen [20–22] because of the increase of complications after the operation.

In the present case, the oldest old patient has been alive with a good condition after the operation. In order to improve the quality of life of the oldest old patients, the practical consideration for esophageal carcinoma should be the individualization of therapeutic protocols, tailoring the extent of resection and inclusion or exclusion of preoperative and postoperative procedures. A curative resection with relatively minimal invasion appears to be mandatory for better prognosis with minimal morbidity and mortality in elderly patients.

## Conclusions

We described a case of successfully treated G-CSF-producing esophageal squamous cell carcinoma in a 92-year-old woman. We assessed the patient’s will and overall condition and chose the best operative method of radical subtotal esophagectomy and could achieve a good clinical course.

## Abbreviations

CRP: C-reactive protein
ESCC: Esophageal squamous cell carcinoma
G-CSF: Granulocyte colony-stimulating factor
SCC-A: Squamous cell carcinoma antigen
